# From Supplements to Sight: Quantifying the Impact of Lutein and Carotenoid on Age‐Related Macular Degeneration—A Systematic Review and Meta‐Analysis of Randomized Controlled Trials

**DOI:** 10.1155/joph/2155378

**Published:** 2026-04-22

**Authors:** Wei-Xiang Wang, Chen-Chi Wang, Wei-Cherng Hsu, Yi-Jie Peng

**Affiliations:** ^1^ Department of Education, Taipei Tzu Chi Hospital, Buddhist Tzu Chi Medical Foundation, New Taipei, Taiwan, tzuchi.com.tw; ^2^ Department of Education, China Medical University Hospital, Taichung, Taiwan, cmu.edu.tw; ^3^ Department of Ophthalmology, Taipei Tzu Chi Hospital, Buddhist Tzu Chi Medical Foundation, New Taipei, Taiwan, tzuchi.com.tw

**Keywords:** age-related macular degeneration, carotenoid, lutein, meta-analysis, systematic review, zeaxanthin

## Abstract

**Purpose:**

To quantify the effects of lutein‐containing supplementation on structural and functional visual outcomes in patients with age‐related macular degeneration (AMD), with particular focus on disease stage and treatment exposure.

**Methods:**

A meta‐analysis of randomized, placebo‐controlled trials was conducted. Nine RCTs involving 860 participants were included. Eligible studies evaluated oral lutein alone or in combination with zeaxanthin or epilutein and reported pre‐ and post‐treatment measurements of macular pigment optical density (MPOD) and best‐corrected visual acuity (BCVA). Random‐effects models were applied to calculate pooled effect sizes using Hedges’ g. Subgroup and meta‐regression analyses were performed to explore stage‐specific responses and dose‐duration associations.

**Results:**

Across the 9 RCTs, lutein‐containing supplementation significantly improved MPOD (Hedges’ g = −0.589, *p* < 0.001) and BCVA (Hedges’ g = −0.827, *p* = 0.001). Improvements were predominantly observed in early‐stage AMD, whereas no statistically significant benefit was detected in late‐stage disease. Lutein monotherapy demonstrated greater visual benefit than combination regimens. Meta‐regression analyses revealed significant positive associations between treatment effect and both supplementation duration and total lutein exposure. Contrast sensitivity and serum lutein concentrations also improved significantly.

**Conclusion:**

Lutein‐based supplementation is associated with measurable structural and functional visual benefits in early‐stage AMD. Treatment effects appear dose‐ and duration‐dependent, while evidence in late‐stage AMD remains limited. These findings support early intervention strategies and warrant further investigation into long‐term therapeutic impact.

**Summary**
 This meta‐analysis demonstrates that lutein and carotenoid supplementation significantly enhances MPOD and visual acuity in patients with AMD, especially with higher doses and extended use. Limited efficacy was found in late‐stage AMD. These findings suggest potential benefits of early supplementation in AMD.

## 1. Introduction

Age‐related macular degeneration (AMD) remains one of the leading causes of irreversible central vision loss among individuals over 65 years of age worldwide. As population aging accelerates globally, the burden of AMD is expected to increase substantially in the coming decades [1]. The disease primarily involves degeneration of the macula, where cumulative oxidative stress and chronic inflammation contribute to dysfunction of the retinal pigment epithelium and progressive photoreceptor loss [2]. Clinically, AMD is categorized into early, intermediate, and late stages according to structural features such as drusen formation and pigmentary abnormalities. While intravitreal antivascular endothelial growth factor (anti‐VEGF) therapy has revolutionized the treatment of neovascular AMD, newer complement inhibitors such as avacincaptad pegol offer emerging options for geographic atrophy [3, 4]. However, these approaches require repeated invasive injections and are associated with considerable economic burden and procedural risks [5, 6]. Importantly, no current intervention restores lost retinal structure once advanced damage has occurred. Therefore, preventive or early‐stage disease‐modifying strategies remain of substantial clinical interest [7].

Among nutritional approaches, lutein and zeaxanthin have attracted particular attention. These xanthophyll carotenoids are naturally present in leafy green vegetables, fruits, and egg yolks and selectively accumulate within the macula to form macular pigment [8]. Functionally, they absorb short‐wavelength blue light and exert antioxidant effects, thereby reducing oxidative stress–induced retinal injury [9, 10]. Experimental and clinical studies have suggested that increased macular pigment levels may support visual performance and potentially attenuate AMD progression [11–13]. Some findings from the Age‐Related Eye Disease Study 2 (AREDS2) indicated that lutein and zeaxanthin supplementation significantly reduced the risk of progression to late‐stage AMD [14]. Notably, beta‐carotene (used in the original AREDS formula) was replaced by lutein and zeaxanthin because it doubled the risk of lung cancer among smokers [15]. Nevertheless, the efficacy of lutein and carotenoid supplementation in AMD remains debated across trials, especially regarding improvements in macular pigment optical density (MPOD) and best‐corrected visual acuity (BCVA).

To address these uncertainties, we performed a systematic review and meta‐analysis of randomized, placebo‐controlled trials evaluating lutein‐containing supplementation in patients with AMD. We aimed to quantify their effects on MPOD, BCVA, and CS and to explore potential modifiers such as disease stage, supplementation duration, and total lutein dosage.

## 2. Methods

### 2.1. General Guidelines

This systematic review and meta‐analysis was conducted in accordance with the PRISMA 2020 reporting guidelines [16]. The review protocol was prospectively registered prior to data extraction (registration number: INPLASY2024100105) [17]. Because this study synthesized data from previously published trials, institutional review board approval and informed consent were not required.

### 2.2. Inclusion and Exclusion Criteria

We included randomized, placebo‐controlled clinical trials enrolling individuals diagnosed with AMD by ophthalmologists. Eligible studies evaluated oral supplementation containing lutein alone or lutein combined with zeaxanthin or epilutein and reported quantitative pre‐ and post‐intervention outcomes for MPOD and BCVA. No restrictions were imposed on participant age or supplementation duration.

Trials were excluded if they were nonrandomized, lacked a placebo comparison group, did not involve lutein‐containing supplementation, or administered additional antioxidant combinations (e.g., fish oil, multivitamins, and zinc formulations) that could confound the isolated effects of carotenoids.

### 2.3. Search Strategy and Study Selection

A comprehensive literature search was independently performed by two investigators across PubMed, Embase, Cochrane CENTRAL, Web of Science, and ClinicalTrials.gov. The search strategy combined controlled vocabulary and free‐text terms related to carotenoids (“lutein,” “zeaxanthin,” “carotenoid”) and AMD (“AMD,” “age‐related maculopathy”) together with trial design filters (“randomized,” “placebo,” “randomized controlled trial”).

All databases were searched from inception through August 18, 2024. Reference lists of relevant systematic reviews were manually screened to identify additional eligible studies. Disagreements during study selection were resolved through discussion with a third reviewer.

### 2.4. Evaluation of Methodological Quality

Methodological quality was evaluated using the revised Cochrane risk‐of‐bias tool (version 2; RoB 2, London, UK) for randomized trials [18]. Domains assessed included the randomization process, deviations from intended interventions, completeness of outcome data, measurement of outcomes, and selective reporting. Each study was categorized as having low risk, some concerns, or high RoB.

### 2.5. Data Extraction and Management

Two reviewers independently extracted study‐level data, including demographic characteristics, AMD stage, supplementation regimen (formulation, dosage, duration), comparator details, and reported outcomes. When multiple follow‐up time points were available, data from the final reported visit were used for pooled analyses to maximize treatment exposure. For studies reporting standard errors (SE), corresponding standard deviations (SD) were derived using the formula SD = SE × √N. When paired analyses were presented without explicit SD values, effect sizes were calculated according to established methods described in the Cochrane Handbook for Systematic Reviews of Interventions.

The primary endpoints were treatment‐associated changes in MPOD and BCVA. Secondary outcomes included contrast sensitivity (CS) and serum lutein concentration. Given the heterogeneity in MPOD measurement techniques—ranging from heterochromatic flicker photometry and flicker‐based psychophysical testing to fundus autofluorescence and reflectance imaging—we standardized continuous outcomes using Hedges’ g to allow cross‐study comparison [19].

### 2.6. Statistical Analyses

Because variability in supplementation regimens, participant characteristics, and measurement techniques was anticipated, pooled estimates were calculated using a random‐effects model [20]. Statistical analyses were conducted using Comprehensive Meta‐Analysis software (Biostat, Englewood, NJ, USA). A two‐tailed *p* value < 0.05 was considered statistically significant.

Effect sizes were expressed as Hedges’ g with corresponding 95% confidence intervals (CIs). Conventional thresholds of 0.2, 0.5, and 0.8 were interpreted as small, moderate, and large effects, respectively [21]. Statistical heterogeneity was evaluated using Cochran’s Q test and the *I* ^2^ statistic, with *I* ^2^ values of 25%, 50%, and 75% representing low, moderate, and high heterogeneity [22].

Prespecified subgroup analyses were conducted to explore potential effect modifiers. Studies were stratified according to AMD stage (early, late, or mixed‐stage populations) and supplementation regimen (lutein monotherapy versus combination formulations). Meta‐regression analyses were further performed to examine associations between treatment effect and supplementation duration as well as total cumulative lutein exposure (daily dose × duration). Sensitivity analyses were conducted by sequentially omitting individual studies to assess the stability of pooled estimates. Publication bias was evaluated using funnel plot inspection and Egger’s regression test, following recommendations from the Cochrane Handbook [21].

## 3. Results

### 3.1. Study Selection and Characteristics

**FIGURE 1:**
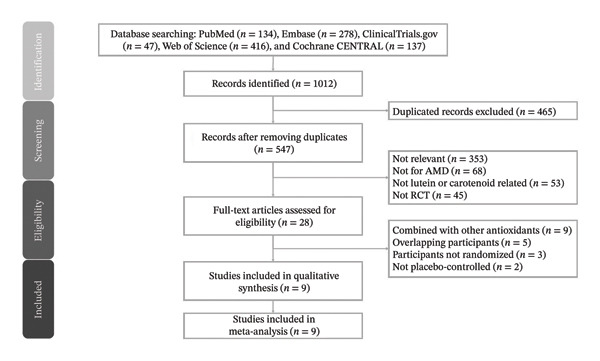
PRISMA 2020 flowchart for the present meta‐analysis. *n*, number; AMD, age‐related macular degeneration; RCT, randomized controlled trial.

The study selection process is illustrated in Figure 1. Following removal of duplicates and screening of titles and abstracts, full‐text articles were assessed for eligibility. Studies were excluded for reasons including nonrandomized design, absence of placebo comparison, inclusion of additional antioxidant combinations, overlapping populations, or failure to report relevant outcome measures. Ultimately, nine randomized controlled trials met all eligibility criteria and were included in the quantitative synthesis.

The nine included RCTs involved a total of 860 participants from Asia, Europe, and America, with a mean age of 71.3 ± 7.4 years and 51.6% men [12, 13, 23–28]. The mean participant age was 71.3 ± 7.4 years, and 51.6% were male. Supplementation duration ranged from 2 to 24 months. Lutein dosages varied between 2 and 20 mg/day, with some regimens incorporating zeaxanthin or epilutein.

MPOD was measured using diverse methodologies, including fundus autofluorescence or reflectance imaging [13, 25, 26, 28, 29], heterochromatic flicker photometry [23, 24], and flicker‐based techniques [27]. AMD stage distribution differed among trials, encompassing early, intermediate, and late disease according to AREDS criteria. Detailed study characteristics are summarized in Table 1.

**TABLE 1:** Study characteristic of the included trials.

Author (Year of Publication)	Country	Age		Sample size (F/M)		Duration (Month)	Dosage (mg/day)	Measurement method for MPOD	AMD stage
		Lutein	Control	Lutein	Control				
Richer et al. 2004 [23]	USA	74 ± 6	76 ± 6	2/27	1/30	12	10 mg L	Heterochromatic flicker photometry	Late

Richer et al.2011 [24]	USA	76 ± 9	74 ± 9	25	10	12	9 mg L + 8 mg Z	Heterochromatic flicker photometer	Early

Weigert et al. 2011 [25]	Austria	Total: 72 ± 9		Total: 66/50		6	1–3 months: 20 mg L 4–6 months: 10 mg L	Fundus reflectance	Early, intermediate, and late

Ma et al. 2012 [26]	China	69 ± 7	69 ± 8	a: 16/10 b: 15/12 c: 15/12	16/11	12	Group a: 10 mg L Group b: 20 mg L Group c: 10 mg L + 10 mg Z	Fundus autofluorescence	Early

Murray et al. 2013 [27]	UK	72 ± 9	69 ± 9	20/16	24/12	12	10 mg L	Flicker‐based technique	Early, intermediate, and late

Huang et al. 2014 [13]	China	69 ± 7	69 ± 8	a: 17/9 b: 13/14 c: 15/12	17/11	24	Group a: 10 mg L Group b: 20 mg L Group c: 10 mg L + 10 mg Z	Fundus autofluorescence	Early

Azar et al. 2017 [29]	Lebanon	75 ± 7	75 ± 7	40/24	34/27	24	5 mg L + 1 mg Z	Fundus autofluorescence	Late

Li et al. 2017 [12]	China	69 ± 7	71 ± 7	52/48	49/51	12	20 mg L	N/A	Early

Forte et al. 2017 [28]	Italy	Total: 64 ± 3		13/8	9/7	2	2 mg L + 8 mg epilutein	Fundus reflectance	Early

*Note:* M = male; F = female; L = lutein; Z = zeaxanthin; AMD = age‐related macular degeneration.

Abbreviations: MPOD = macular pigment optical density; N/A = not available.

### 3.2. Quality Assessment of the Included Studies

**FIGURE 2:**
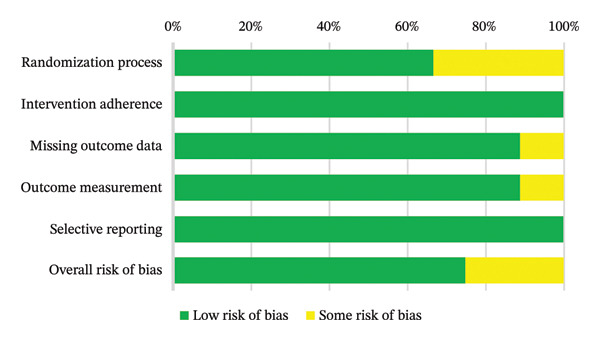
Summary of quality assessment of the included studies in the study using Cochrane risk of bias 2 tool.

Overall methodological quality was considered acceptable. Six studies (66.6%) were judged to have low RoB, whereas three (33.3%) were rated as having some concerns. No study was categorized as high risk (Figure 2). Concerns primarily related to insufficient reporting of allocation procedures, incomplete outcome data in small samples, or limited description of outcome measurement methods. A comprehensive domain‐level assessment is provided in Supporting Table S1.

### 3.3. Primary Outcome and Subgroup Analysis: MPOD and BCVA

**FIGURE 4:**
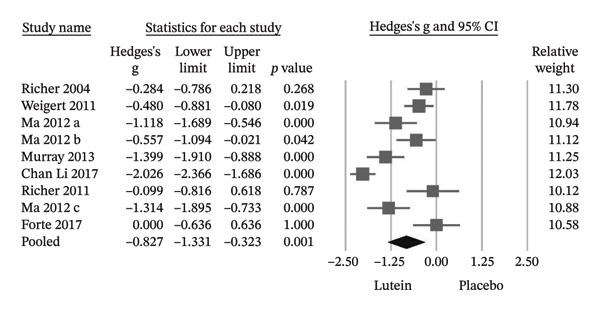
The lutein group showed a statistically significant improvement in BCVA compared to the placebo group. Cl, confidence interval.

Pooling data from nine RCTs demonstrated a significant improvement in MPOD following lutein‐containing supplementation compared with placebo (Hedges’ g = −0.589; 95% CI −0.839 to −0.340; *p* < 0.001), with substantial heterogeneity observed (*I* ^2^ = 70.6%) (Figure 3(a)). To evaluate robustness, a leave‐one‐out sensitivity analysis was performed. Sequential exclusion of individual trials did not materially alter the direction or statistical significance of the pooled estimate, indicating stability of the MPOD findings (Figure 3(b)). Seven of the nine trials reported statistically significant improvements in BCVA in the lutein group relative to placebo. The pooled effect size confirmed a significant benefit (Hedges’ g = −0.827; 95% CI −1.331 to −0.323; *p* = 0.001) (Figure 4).

FIGURE 3(a) Among these 9 RCTs, lutein supplementation resulted in a statistically significant improvement in MPOD. (b) The sensitivity analyses showed that the efficacy of lutein supplementation in improving MPOD remained consistently statistically significant across all analyses. Cl, confidence interval.

**Figure:**
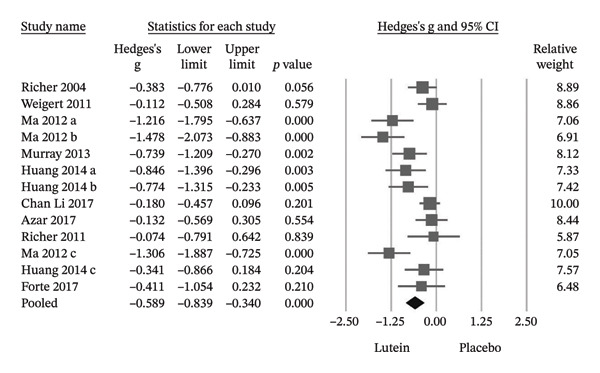
(a)

**Figure:**
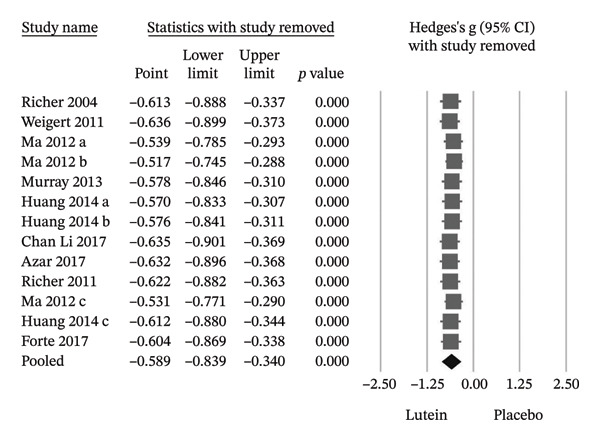
(b)

When stratified by disease stage, treatment effects differed. In early‐stage AMD, supplementation was associated with significant improvements in both MPOD (Hedges’ g = −0.725; *p* < 0.001) and BCVA (Hedges’ g = −0.880; *p* = 0.01) (Figures 5(a) and 5(b)). In contrast, no statistically significant changes were observed in late‐stage AMD for MPOD (Hedges’ g = −0.260; *p* = 0.425) or BCVA (Hedges’ g = −0.284; *p* = 0.732). Similarly, trials including mixed early‐to‐late stage populations did not demonstrate significant pooled effects (MPOD: Hedges’ g = −0.414; *p* = 0.209; BCVA: Hedges’ g = −0.931; *p* = 0.109) (Figures 5(a) and 5(b)).

FIGURE 5(a) In the subgroup analysis of AMD, patients in the early stage group showed significant improvements in MPOD, but not significant in the late and early‐to‐late stage group. E, early; L, late. (b) The early stage group showed significant improvements in BCVA, but not significant in the late and early‐to‐late stage group. E, early; L, late; E to L, early‐to‐late; Cl, confidence interval.

**Figure:**
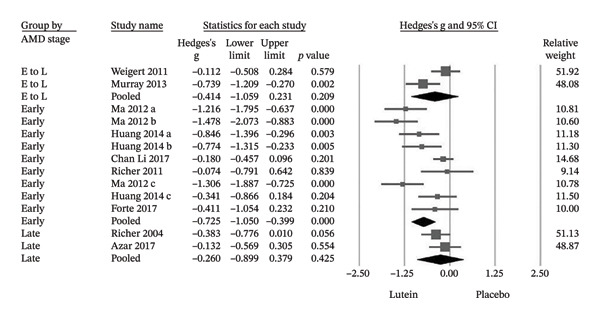
(a)

**Figure:**
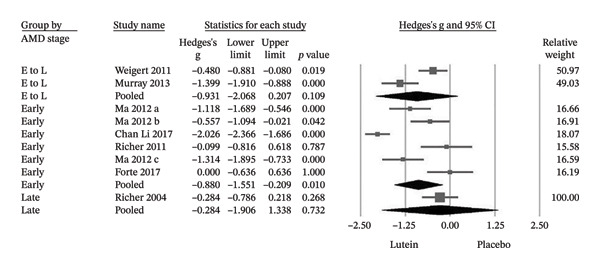
(b)

When analyzed according to regimen composition, lutein monotherapy was associated with significant improvements in MPOD (Hedges’ g = −0.606; *p* < 0.001) and BCVA (Hedges’ g = −0.985; *p* = 0.002) (Supporting Figure S1). By comparison, combination formulations (lutein plus zeaxanthin or epilutein) showed a significant benefit for MPOD (Hedges’ g = −0.551; *p* = 0.029), whereas the effect on BCVA did not reach statistical significance (Hedges’ g = −0.484; *p* = 0.291) (Supporting Figure S1).

### 3.4. Secondary Outcome: CS and Serum Lutein Level

**FIGURE 6:**
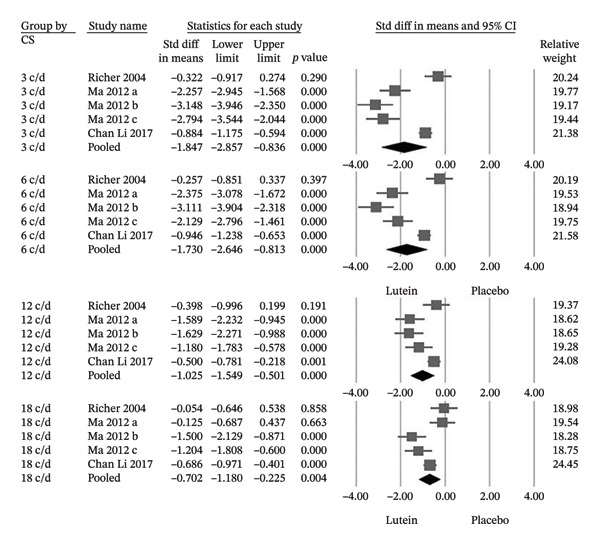
The lutein group showed statistically significant improvement in CS at 3, 6, 12, and 18 c/d compared to the placebo group. CS, contrast sensitivity; c/d, cycles per degree; Cl, confidence interval.

**FIGURE 7:**
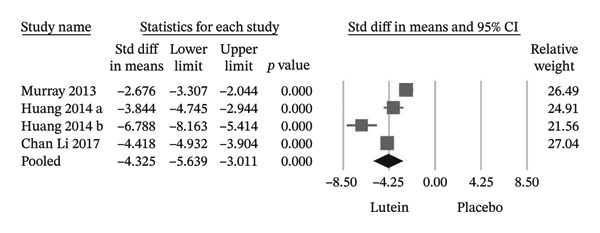
The lutein group demonstrated a significant increase in serum lutein levels. Cl, confidence interval.

Among the trials reporting CS, supplementation resulted in significant improvements at spatial frequencies of 3, 6, 12, and 18 cycles per degree, with the largest effect sizes observed at lower spatial frequencies (Hedges’ g values −1.847, −1.730, −1.025, and −0.702, respectively) (Figure 6). In addition, three trials demonstrated significant increases in serum lutein concentrations following supplementation (Hedges’ g = −4.325; 95% CI −5.639 to −3.011), confirming effective systemic absorption (Figure 7).

### 3.5. Meta‐Regression and Publication Bias

**FIGURE 8:**
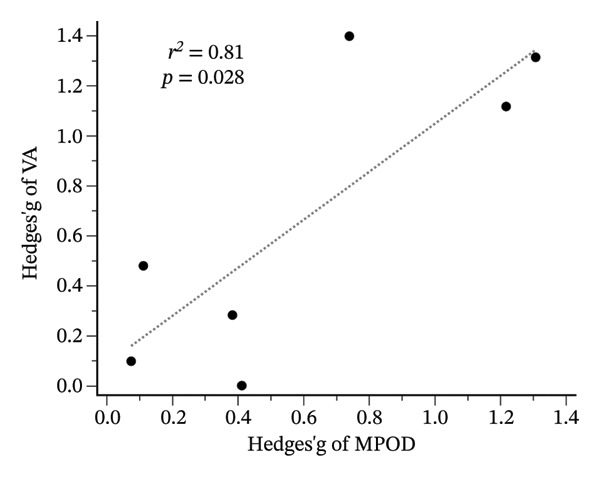
Meta‐regression for Hedges’g of MPOD and BCVA reported positive significant correlation. MPOD, macular pigment optical density; BCVA, best‐corrected visual acuity; *r* ^2^, coefficient of determination; *p*, *p* value.

Meta‐regression analysis revealed a significant association between improvements in MPOD and BCVA (*r* ^2^ = 0.81; *p* = 0.028) (Figure 8). Longer supplementation duration was significantly correlated with greater improvements in both MPOD (coefficient = −0.0336; *p* < 0.001) and BCVA (coefficient = −0.0829; *p* < 0.001) (Supporting Figure S2). Similarly, total cumulative lutein exposure (daily dose × duration) demonstrated significant dose–response relationships for MPOD (coefficient = −0.0001; *p* < 0.001) and BCVA (coefficient = −0.0002; *p* < 0.001) (Supporting Figure S2). Visual inspection of funnel plots suggested asymmetry for MPOD outcomes, and Egger’s regression test indicated potential publication bias (Supporting Figure S3).

## 4. Discussion

The present meta‐analysis included 9 RCTs until the year 2024, involving 860 participants across different AMD stages. It found that lutein and carotenoid supplementation improved MPOD, BCVA, and CS. These results are more robust than those of the previous meta‐analyses, because we excluded the interactions of fish oil or antioxidants and included only RCTs [30–33]. Sensitivity analysis consistently showed support for the stability of the results. Subgroup analyses revealed that lutein supplementation significantly improved MPOD and BCVA in early‐stage AMD but not in late‐stage or mixed‐stage groups. Similarly, the lutein‐only group showed significant benefits for MPOD and BCVA, while no significant BCVA benefit was observed when combined with other carotenoids, likely due to the smaller sample size of the combination group. Serum lutein levels increased significantly following supplementation, confirming effective systemic absorption. The meta‐regression analysis demonstrated significant improvements in MPOD and BCVA with longer supplementation duration and higher total lutein dosage, supporting a dose‐ and time‐dependent functional benefit.

Lutein and other carotenoids act as macular pigments that maintain macular function and filter blue light to protect the retina, thereby improving image quality and leading to better BCVA. Lutein also inhibits inflammatory mediators, eliminates reactive oxygen species, and reduces the expression of the nicotinamide adenine dinucleotide phosphate oxidase subunit Nox4 [34]. Lutein supplementation significantly increases total antioxidant capacity and antioxidant enzyme levels in vivo [35]. Lutein protects human retinal pigment epithelial cells (ARPE‐19) from oxidative stress and prevents aging in vitro [36]. Our findings suggest that lutein possesses antioxidant properties and may slow AMD progression, improving outcomes including MPOD, BCVA, and CS. Among these, MPOD, the most important outcome, was detailedly measured using different methods including fundus reflectance, autofluorescence, and heterochromatic flicker photometry. To avoid measurement bias, we analyzed the changes in MPOD before and after carotenoid supplementation and expressed the results with Hedges’ g. Notably, the meta‐regression revealed a significant positive correlation between MPOD and BCVA.

Although the significant improvement in BCVA among early‐stage AMD patients may seem unexpected given their near‐normal baseline vision, the change was small and likely reflects preservation of visual function rather than true acuity gains. Prior studies have suggested that CS enhancement, glare reduction, or prevention of age‐related decline may explain these effects [13, 26]. In addition, lutein supplementation may help maintain baseline visual acuity, whereas the placebo group experiences slight age‐related visual decline, resulting in a net positive difference. We also reviewed several previous meta‐analyses. In 2016, Ma et al. reported that carotenoid supplementation could improve MPOD in both patients with AMD and healthy individuals, and a dose‐response relationship was reported [32]. In 2014, Liu et al. and Wang et al. reported that carotenoid supplementation could improve MPOD, BCVA, and CS in patients with AMD, and similar results were reported by Liu et al. [30, 31, 33]. In 2022, Liu et al. found in a subgroup analysis that a high‐dose (20 mg/day) and long‐term (> 6 months) lutein supplementation administration may offer better efficacy for patients with AMD [37].

Despite these insights, the past meta‐analyses did not exclude confounding factors in the intervention groups nor clarify the benefits of carotenoids across different AMD stages. Therefore, we excluded studies that included other antioxidants, fish oils, or zinc ions and focused only on carotenoids to minimize selection bias. To understand prognosis at different AMD stages, we included RCTs with patients in early, intermediate, or late‐stage AMD and analyzed different prognostic outcomes, including MPOD, BCVA, and CS. We found that lutein and zeaxanthin significantly improved MPOD and BCVA in early‐stage AMD; however, no significant effect was observed in late‐stage AMD. This result is similar to the previous RCTs, and some AREDS2 studies have also reported that lutein and zeaxanthin do not decrease the risk of progression to late‐stage AMD [26, 38]. The AREDS2 formulation (lutein 10 mg and zeaxanthin 2 mg/day) serves as a widely accepted clinical reference, and our meta‐regression suggests a dose–response trend favoring higher doses and longer duration, particularly in early‐stage AMD [39, 40]. Importantly, lutein and zeaxanthin were incorporated into the AREDS2 formula because of their lower associated risk of lung cancer in smokers compared with beta‐carotene [38].

Although this study revealed the significant benefits of lutein and carotenoid supplementation, several limitations need to be addressed. First, substantial heterogeneity was observed across the included studies, partly attributable to differences in ethnicity, follow‐up duration, and heterogeneous carotenoid regimens (lutein 2–20 mg/day). As this meta‐analysis was not a dose‐finding study, it does not allow determination of an optimal supplementation dose. Sensitivity analyses and meta‐regression were conducted to address heterogeneity. Second, the trials varied in patient characteristics (such as age and sex) and in the methods used to measure MPOD and BCVA. These differences may have influenced the efficacy of the carotenoid supplementation. However, we mitigated this issue by using Hedges’ g to standardize outcomes across studies. Third, although our meta‐regression analyzed the relationship between supplementation duration and improvements in MPOD and BCVA, AMD is a chronic disease that may require much longer follow‐up, such as the 5 years tracked in the AREDS2 study [38]. However, the RCTs included in our meta‐analysis only tracked outcomes for 2–24 months. Despite these limitations, our meta‐analysis demonstrated the significant benefits of lutein and carotenoid supplementation and provides a more detailed explanation of their efficacy across different AMD stages.

## 5. Conclusion

Lutein and carotenoid supplementation significantly improved MPOD, which helps protect the retina by reducing blue‐light‐induced damage to visual function. It also enhances the antioxidative environment, leading to a stable improvement in BCVA and CS, allowing better visual performance under low‐light or high‐glare conditions. This study demonstrates significant benefits of lutein supplementation in early‐stage AMD, while the lack of significance in late‐stage AMD may be attributed to the limited number of articles analyzed. Future research should further evaluate the long‐term effects of lutein and carotenoid supplementation in the management of late‐stage AMD.

## Funding

This research did not receive any specific grant from funding agencies in the public, commercial, or not‐for‐profit sectors.

## Conflicts of Interest

The authors declare no conflicts of interest.

## Supporting Information

Table S1. PRISMA 2020 Checklist.

Table S2. Detailed quality assessment of included studies using Cochrane risk of bias 2 tool. S, some risk of bias; L, low risk of bias; RoB, risk of bias.

Figure S1. (a, b) In the subgroup analysis of the lutein regimen, the lutein‐only group showed significant improvements in both MPOD and BCVA. However, the compound group demonstrated significant improvement in MPOD but no significant difference in BCVA.

Figure S2. Both MPOD and BCVA showed significant positive correlations in the meta‐regression for supplementation duration and total dose (daily dose × duration).

Figure S3. Funnel plot for MPOD showed asymmetric distribution with *p* value of the Egger’s test less than 0.5, revealing publication bias.

## Supporting information


**Supporting Information** Additional supporting information can be found online in the Supporting Information section.

## Data Availability

The authors have nothing to report.
